# Proving of Tussilago Petasites

**Published:** 1889-08

**Authors:** E. W. Berridge

**Affiliations:** London


### PROVING OF TUSSILAGO PETASITES.

E. W. Berridge, M. D., London.

The plants were gathered when flowering, at beginning of 1865, at Great Malvern, Worcestershire, by the late Dr. J. R. Croker. The fresh roots were sliced and macerated for three months in a mixture of two parts of water to one of alcohol, and pressed.

A description of the plant is given in Bentham's *Hand-book of the British Flora*, 1865, p. 415, fig. 492.

*First proving.*—August 16th, 1865, I took twelve drops of the tincture in water, at 11.30 A. M.

1.40 P. M., slight, dull throbbing pain in abdomen for a few minutes.

2 P. M., stinging, burning sensation on anterior part of dorsum of tongue, as from pepper, lasting two hours, then decreasing; re-appeared slightly at intervals for remainder of day.

August 17th.—Flat, disagreeable taste on tongue on rising in morning.

At 7 A. M. took a drachm of the tincture in water.

7.30 A. M., slow, throbbing pains in abdomen, below umbilicus, for a minute.

8.40 A. M., very small stool, rather difficult, but painless (doubtful symptom).

Anterior part of dorsum of tongue feels sore, as if it had been scraped, the first part of morning.

At 10.07 A. M. took a drachm of tincture in water, and at 12.45 P. M. took two drachms in water.

August 18th.—Soreness of tip of tongue on rising in morning.
7.50 A. M., took half an ounce of tincture in water, and at
12.40 P. M., took one ounce in water.
1 P. M., great sleepiness after long walking.
6.30 P. M., soreness of tip of tongue.
In afternoon scanty stool (doubtful symptom).

August 19th.—About 7 A. M., soreness of tip of tongue. Took an ounce of tincture in water.

Evening, after 9 P. M., felt tired, with disinclination to be spoken to; voices seemed unpleasantly loud, with slight feeling of faintness.

August 20th.—No stool (doubtful symptom).

*Second proving.*—Jan. 11th, 1888.—Dr. David Wilson told me that he had a tickling at throat-pit, causing cough. When cough was bad it caused a splitting feeling in brain at occiput. He had also a feeling on tongue as if burnt by pepper, extending downward. I mentioned that *Petasites* had caused the last symptom on myself, and he at once took, at 12.25 P. M., five globules of the twelfth potency, the only one he had. It had been prepared by a homœopathic chemist. The following new symptoms then ensued:

Considerable headache on vertex, spreading along coronal region, like a very slight burning under the scalp, it then passed on to tension in scalp of coronal region, in four minutes.

In five minutes the same tensive aching slightly spread over thorax.

Directly afterward, tendency to sneeze, from irritation in left nostril.

In a few minutes more, slight transient, internal creeping in left ear, followed by increased headache on vertex; the headache also slightly affected the upper segment of eyes with a sort of pressure. Tickling in throat better.

1.30 P. M. All the pathogenetic symptoms gone. The burning on tongue, which he had had for five or six years, is much better.

Peppery sensation on tongue belongs to *Cann-ind.*, *Laches.*, *Mercurius*, *Mezereum*, *Opium*. The first two are given in Allen's *Repertory*, p. 1204, under "Pepper," the last three at p. 1200, under "Burning."

Of course, these last three ought all to have been placed under the rubric of "Pepper" as well as "Burning."

